# Digital technologies to support people living with dementia in the care home setting to engage in meaningful occupations: protocol for a scoping review

**DOI:** 10.1186/s13643-021-01715-4

**Published:** 2021-06-21

**Authors:** Nicholas Luscombe, Sarah Morgan-Trimmer, Sharon Savage, Louise Allan

**Affiliations:** 1 The Centre for Research in Ageing and Cognitive Health (REACH), University of Exeter Medical School, St. Luke’s Campus, Heavitree Road, Exeter, EX1 2LU UK; 2 Institute of Health Research, College House, University of Exeter, St Luke’s Campus, Heavitree Road, Exeter, EX1 2LU UK; 3 School of Psychology, The University of Newcastle, University Drive, Callaghan, NSW 2308 Australia

**Keywords:** Digital intervention, Tailored activities, Social engagement, Social connectedness, Isolation, Person-centred care, Long-term care

## Abstract

**Background:**

People living with all stages of dementia should have the opportunity to participate in meaningful occupations. For those living in care homes, this may not always occur and residents may spend significant parts of the day unengaged, especially those living with more advanced dementia. Digital technologies are increasingly being used in health care and could provide opportunities for people living with dementia (PLWD) in care homes to engage in meaningful occupations and support care staff to provide these activities. With technology advancing at a rapid rate, the objective of this scoping review is to provide an up-to-date systematic map of the research on the diverse range of digital technologies that support engagement in meaningful occupations. In particular, focus will be given to barriers and facilitators to inform future intervention design and implementation strategies, which have not yet been clearly mapped across the full range of these digital technologies.

**Method:**

A scoping review will be conducted to systematically search for published research using a comprehensive search strategy on thirteen databases. Published, peer-reviewed studies that focused on PLWD in the care home setting and assessed any form of digital technology that supported a meaningful occupation will be included. All methodologies which meet the criteria will be included. Data will be extracted and charted to report the range of digital technologies, underlying mechanisms of action, facilitators and barriers to implementation.

**Discussion:**

Mapping the range of technologies to support PLWD to engage in meaningful occupations will identify gaps in research. The systematic search will include a diverse range of technologies such as software to enhance care planning, tablets devices, smartphones, communication robots and social media platforms, rather than focussing on a specific design or interface. This will enable comparison between mechanisms of action, barriers and facilitators to implementation which will be useful for future research and intervention design.

**Trial registration:**

Open Science Framework 10.17605/OSF.IO/7UDM2

**Supplementary Information:**

The online version contains supplementary material available at 10.1186/s13643-021-01715-4.

## Background

The majority of care home residents (69%) live with dementia [1] and a lack of tailored activities [2, 3] has been reported for these residents. This is despite national guidelines recommending that people living with dementia (PLWD) should be offered activities tailored to their preferences [4]. Barriers include limited resources, understaffed environments and time constraints [5, 6]. PLWD in care homes are at risk of spending significant parts of the day socially unengaged [7, 8]; one study identified that residents spent as little as 10% of the day socially engaged [9]. Loneliness and isolation can lead to feelings of unhappiness and even cause fear for some PLWD [10]. A lower mortality rate has been reported in care home residents involved in social activities [11] and a positive relationship between social interaction and a higher quality of life has also been reported in PLWD [12, 13]. Engaging in activities that are meaningful or personalised to the PLWD have been reported to reduce agitation [14, 15], improve affect and engagement [16], although these benefits have not been consistently reported between reviews [17]. In addition, these interventions, by their nature, often involve increased social interaction which may be acting as a confounding factor [15–18].

Terminology regarding tailoring activities is inconsistent in the literature. The term occupation is a more comprehensive definition as this includes purposeful activities and incorporates self-identity [19]. For the purpose of this review, the term ‘meaningful occupation’ will be used, which Travers et al. describes as any of: “a wide range of activities …[that] is somehow significant to, or valued by, the person and provides enjoyment, a sense of purpose, belonging or achievement” [15]. This will include activities involving social interaction [18] as these can be a meaningful occupation for PLWD and provide a sense of inclusion, value and contribution [6, 20, 21].

### Digital technology in care homes

A wide-range of technologies are available to support care homes such as: telemedicine, tele monitoring, digital care records and technologies for leisure [22, 23]. UK data on the uptake of digital technology in care homes is limited. Research in the USA has demonstrated rising trends in the adoption of technology in care homes [24] but having access to technology does not necessarily mean it is used to its fullest capabilities [25]. Previous reviews have provided a broad overview of technologies developed for PLWD [22, 26, 27] although some have included technologies unrelated to the engagement in meaningful occupations [22, 26, 27], or with limited focus on the care home setting [26, 28]. The care home setting is a distinct environment with many contextual factors that need to be considered in order to successfully implement an intervention [5, 6]. More recently, Goodall et al. [29] undertook a systematic review of technologies used to provide meaningful activities for PLWD. As with previously mentioned reviews, the majority of research was conducted in the community. Reported benefits of individualised technologies included improvements in mood, behaviour, self-identification and relationships [29]. The authors highlight some potential challenges in the application of technology in the care home setting; the majority of the technologies required assistance from another person, time constraints for care staff and potentially a higher proportion of residents having more advanced dementia than in the community [29]. Although a breakdown of each study’s barriers and facilitators specific to the care home setting has not been provided.

Mobile technologies, such as tablet devices with touchscreen interfaces (i.e. iPads), have become more widespread in recent years and could be used to support PLWD [30]. Hung et al. [31] specifically reviewed the application of touchscreen technology to support social connections for PLWD in care environments such as hospitals and care homes. Not all studies, however, solely focused on PLWD [32, 33] or the care home setting [34–36]. Reported benefits of using a touchscreen device included improved engagement, behaviours and quality of life [31]. Challenges to using a touchscreen device included the ability of residents to physically hold the device, reflective glare, internet connectivity, battery life and concerns with residents data being kept confidential [31]. Importantly, only devices with touchscreen interfaces were included in this review, excluding a range of other digital approaches. When designing new technology, it is important to consider the barriers and facilitators to all types of interface.

The review by Neal et al. [37] is the only review to our knowledge that has focused on the use of technology to promote meaningful engagement for PLWD specifically in the care home setting. Two areas of research were described: robotics (*n* = 14) and multimedia devices (*n* = 6) [37]. The majority of research (*n* = 12) focused on ‘robopets’ which are animal-like robots that mimic an animal in appearance and behaviour [38, 39], for example AIBO a dog-like robot [40]. Only six studies which utilised non-robotic technologies [34, 41–45] were identified by the review [37], which appears to show a relatively limited range of research on multimedia technologies. Neal et al. suggest for people with more advanced stages of dementia, only technologies which facilitate social interaction will provide meaningful engagement [37]. Although, they also conclude that there is limited research which has explored the perspectives of PLWD when using a technology, as the majority of studies focus on the perspectives of care staff or family [37]. Facilitators and barriers to using and implementing these technologies were not explored in detail in this review.

### Rationale and contribution

This review aims to systematically map the published research on the range of digital technologies that have been used to support the provision of meaningful occupation for PLWD, specifically in the context of the care home setting. This review aims to extend beyond other reviews and summarise the reported facilitators, barriers and user perceptions across a broad range technology designs which have not been previously synthesised. This will also include charting data on the development stages of the technology, such as user centred design.

A possible limitation in previous reviews [29, 31, 37] is that older studies prior to 2005 have not been included. Digital technologies have developed at a rapid pace in recent years; however, older technologies could still provide valuable insights. This will be addressed by our scoping review as no limitations for the date of publication will be set.

A comprehensive search strategy has been developed to identify a broader range of technologies. This will include search terms to explore research on the use of electronic care records to facilitate meaningful occupations, which has not been explored in previous reviews. Additionally, we have developed a broad list of search terms on the topic of social connectedness and we will include sociology databases to identify socially interactive technologies that maintain connections with others. This is an especially important area at this time because of the ongoing impact of COVID-19 on people living in care homes and the resulting loss of social contact they may have with friends and family. This will focus on technologies that directly facilitate social interaction between two humans (i.e. video calls, Skype or email) rather than activities which are deemed social because of their application in a group setting, such as robopets. A broad range of technology interfaces will be included rather than focussing on specific types of interface as this will enable a comparison of facilitators and barriers between technology designs.

### Research questions and objectives

Two proposed questions will be answered by this review. Firstly, “What types of digital technologies have been used to support the provision of meaningful occupation for PLWD in the care home setting?” Additionally, “What are the reported mechanisms of action, methods of delivery, facilitators and barriers?”

## Objectives:

1. Systematically map the published research on digital technologies that have been used to support the provision of meaningful occupation for PLWD in the care home setting.
2. Summarise the interventions: mechanism of action, components and delivery in the care home setting.
3. Identify reported facilitators and barriers to implementation.

A scoping method has been chosen as this will allow a systematic search to be undertaken to identify the broad range of digital technologies which can then be mapped [46, 47]. The existing literature encompasses an assortment of methodologies. Outcomes from both quantitative and qualitative research are valuable for informing the design of digital interventions [48]; therefore, a scoping method will enable inclusion of all methodologies. In addition, the scoping method does not involve appraising the quality of evidence; therefore, an overview of all research can be presented, including more detailed findings to meet the research objectives [46].

## Methods

The review protocol has been registered with the Open Science Framework (OSF) database (OSF.IO/7UDM2). The review will be reported in accordance with the guidance provided in the Preferred Reporting Items for Systematic reviews and Meta-Analyses extension for scoping reviews (PRISMA-ScR) [49] (see Additional file 1: PRISMA-ScR checklist). The review will be conducted following the methodology described by Arksey et al. [46] and guidance from the Joanna Briggs Institute (JBI) Reviewers Manual [50].

### Information sources and search strategy

Following the guidance from the JBI the search strategy will follow three stages [50]. Firstly, prior review of the relevant literature by the principal researcher (NL) has informed the initial list of search terms and search strategy, with additional input and revision from the research team and an information specialist. MEDLINE (Ovid) and the Cumulative Index to Nursing and Allied Health Literature (CINAHL) (EBSCO) databases have been used to pilot the search strategy and relevant articles were reviewed to identify further key words for inclusion. Additional key words have been identified following review of articles in personal libraries and relevant reviews in related areas.

In the second stage, the comprehensive list of search terms will be used to search for published peer-reviewed literature using the following electronic databases: MEDLINE (Ovid), APA PsycInfo (Ovid), Embase (Ovid), HMIC Health Management Information Consortium (Ovid), Social Policy and Practice (Ovid), Cumulative Index to Nursing and Allied Health Literature (CINAHL complete) (EBSCO), AgeLine (EBSCO), Scopus, Web of Science, Cochrane Library, British Nursing Index (BNI) (Proquest), Sociology collection (Applied Social Sciences Index and Abstracts (ASSIA), Sociological abstracts, Sociology Database) (Proquest), and The Association for Computing Machinery (ACM) Digital Library.

When available Boolean operators will be used, along with modifiers, to ensure variants of the English language are identified and searches are limited to peer-reviewed journals. Broad search terms will be used when databases do not have advanced search functions and no date limits will be applied. A draft search strategy for MEDLINE is provided in Additional file 2. We will perform hand-searching of the reference lists of included studies, relevant reviews or other relevant documents.

### Study selection

Studies identified from the systematic search will be compiled into an Endnote library and duplicates removed. Two reviewers (principal researcher NL and a second reviewer) will independently assess the titles and abstracts against the selection criteria. Any divergence between the two reviewers will be discussed for consensus; if unresolved a third independent reviewer will be consulted. Studies will then undergo full-text review by the principal researcher (NL) and reasons for exclusion will be recorded and reported. A PRISMA flowchart [51] will be used to display the decision process for inclusion.

The inclusion/exclusion criteria (Table 1) have been developed using the Population, Concept and Context framework [50]. The criteria have been applied to a random selection of articles from the pilot searches and modifications were made following discussion with the research team.

**Table 1:** Inclusion and exclusion criteria

	Inclusion criteria	Exclusion criteria
Population	Participants of any age, gender or ethnicity living with dementia, of any type or severity. Studies which included participants without dementia reported the outcomes for PLWD separately.	The study population was not focussed on PLWD or studies with mixed cohorts did not report the outcomes for PLWD separately.
Concepts	Describes the use of a digital technology, defined for this scoping review as an electronic computer-based technology that processes data using binary code.	The intervention studied was not a digital computer-based technology.
	Supports PLWD to engage in a meaningful occupation: -Used for a personalised activity (i.e. personal music playlist). -Specifically tailored to a person’s preferences or abilities. -Facilitated engagement in a meaningful occupation (i.e. care staff use of care note software or predictive algorithms) -Maintained self-identity (i.e. digital life story book) -Maintained connections with others (i.e. social media, video calls, and social robots).	The intervention did not support engagement in a meaningful occupation: -Generic technology which has not been personalised or used to connect with others. -Telemedicine or telecare where the technology is used to provide medical care rather than a meaningful occupation. -Monitoring, tracking or assistive technology for personal care (such as alarm clocks, medication reminders) unless this facilitated a meaningful occupation (such as tracking algorithms to predict and plan meaningful occupations). -Technology used solely for e-learning/training.
Context	The technology was used in a care home setting which includes both care homes with nursing (nursing home) and those without (residential care home).	The study did not specifically focus on the care home setting (such as domiciliary care, day centres or extra care housing services).
	The study was primary research utilising any methodology.	Review of evidence (such as literature reviews, systematic reviews and scoping reviews). Conference paper, editorial or book chapter. Duplicate or correction of no significance.
	Published in a peer-reviewed journal in any year.	Non-peer reviewed.
	A full-text version in the English language is available.	A full-text version in the English language is not available.

### Population

The digital technology was used to support meaningful occupation for people living with dementia, of any type or severity. Participants may be of any age, gender or ethnicity. Studies with mixed cohorts of participants without dementia will only be included if outcomes have been specifically reported for those living with dementia, such as a sub-analysis or case reports. This will include studies where residents may not have been given a formal diagnosis of dementia, but where dementia has been assumed based on the combination of both significant cognitive decline (e.g. low MMSE) and functional impairments.

### Concepts

Describes the use of a digital technology, defined for this scoping review as an electronic computer-based technology that processes data using binary code, to support engagement in a meaningful occupation (as previously defined). Studies will be included if the technology; was used for a personalised activity (i.e. personal music playlist), was specifically tailored to a person’s preferences or abilities, facilitated engagement in a meaningful occupation (i.e. care staff use of care note software or predictive algorithms), maintained self-identity (i.e. life story book) or maintained connections with others (i.e. social media, video calls, social robots).

Technology used for monitoring, tracking or assistive technology for personal care (such as alarm clocks or medication reminders) will not be included unless this facilitated a meaningful occupation (such as tracking algorithms to predict and plan meaningful occupations). Telemedicine or telecare where the technology is used solely to provide medical care rather than a focus on meaningful occupation will not be included. Generic technology with no personalised aspect and e-learning will also be excluded.

### Context

The review will focus on research that has taken place in a care home setting which will be defined as: a service which “provides accommodation, together with nursing or personal care” [52]. This includes care homes with nursing (nursing home) or without (residential care home) [53, 54]. Living in a shared communal environment which is overseen by carers brings significant differences compared to people living in their own homes in the community. Therefore, people living in the community receiving domiciliary care, attending day centres or extra care housing services will not be included.

The review will include studies of any methodology that have been published in a peer-reviewed journal in any year, that are available in full text and English, as sufficient information is required to be able to report on the hypothesized theories and reported barriers.

### Charting the data

Data will be extracted using a charting form (see Additional file 3: Charting form). The charting form, modified from the JBI template [50], has been developed following pilot tests and will be used to extract sufficient detail to ensure the research aims are met and the context of each study is conveyed [46]. Data will be extracted independently by the principal researcher and discussed with the research team. Study authors will be contacted if the data is incomplete or if there is any uncertainty. A detailed description of the technology will be extracted which will include the interventions development stages, components, method of delivery, interface, training and support requirements. In addition, data on the hypothesised mechanism of action and concepts which create a meaningful occupation will be extracted. Data on barriers and facilitators to implementation and user perceptions will be extracted in detail. Outcome measures used by each study (e.g. neuropsychiatric symptoms, social engagement) will also be extracted.

### Collating, summarising and reporting the results

Charted data will be collated and priority will be given to explore the themes which underpin the theory behind the digital intervention, concepts of meaningful occupations, facilitators and barriers to implementation. The narrative will be organised deductively around the underlying concepts of meaningful occupations for PLWD as previously described [15, 20] rather than the design of the technology. This will include summarising the gaps in the evidence [47]. Reported facilitators and barriers will be tabulated and frequency counts reported [50]. Basic numerical data on the range and distribution of the included studies will be summarised with descriptive statistics and presented in tables or charts [46]. In addition, maps will be used to present an overview on the range of technologies along with their underlying theory or concept of meaningful occupation.

## Discussion

For transparency, the review protocol has been registered with the OSF. If any protocol amendments are required when conducting the review, these will be documented in the OSF protocol registration and in the final manuscript. No new data will be collected from participants; therefore, ethical review will not be required. During the iterative process of developing the narrative, consultation will take place with a Patient and Public Involvement (PPI) group and experts in the field.

Once the scoping review is complete, the findings will be disseminated in an open access publication in a peer-reviewed journal. Dissemination plans also include the presentation of findings at relevant conferences and publication in open access repositories.

Potential limitations of this review have been considered. Firstly, as with many reviews, there is the chance relevant studies could be missed by the search strategy. To reduce this risk, a systematic and comprehensive search strategy has been developed following preliminary review of the literature. In addition, hand-searching of the reference lists of relevant documents will be performed. Secondly, the review will include studies utilising all methodologies which could create difficulties when developing a narrative; therefore, researchers with expertise in both quantitative and qualitative methodologies will provide input into the review. Lastly, the concept of meaningful occupations could create operational issues due to its subjective interpretation. To minimise this, the term has been operationalised taking into consideration terms used in previous reviews. During the screening process, it is anticipated that additional time may be required for screeners to discuss any disagreement and if required a third independent screener will be available.

Although possible limitations exist, this review will provide a systematic map of the range of digital technologies used to support the provision of meaningful occupation for PLWD living in care homes. Our review will extend beyond existing reviews by capturing a broader range of technologies and include socially interactive technologies that maintain connections with others. Identifying the gaps in research, summarising the mechanisms of action and barriers to implementation will help identify areas for future research and could be useful to inform the development of future digital interventions.

## Supplementary Information

**Additional file 1.** PRISMA-ScR checklist.

**Additional file 2.** Search strategy for MEDLINE.

**Additional file 3.** Charting form.

## Data Availability

Data sharing is not applicable to this article as no datasets were generated or analysed during the current study.

## Abbreviations

PLWD: People living with dementia
PCC: Person-centred care
PRISMA: Preferred Reporting Items for Systematic reviews and Meta-Analyses
JBI: Joanna Briggs Institute
PPI: Patient and Public Involvement
