# Supplementary material for: Digital technologies to support people living with dementia in the care home setting to engage in meaningful occupations: protocol for a scoping review
Source: Syst Rev. 2021 Jun 21;10:179. doi: 10.1186/s13643-021-01715-4 (PMC8214930; doi:10.1186/s13643-021-01715-4)
Supplement: Supplementary file 2 — Additional file 2. Search strategy for MEDLINE. [file 13643_2021_1715_MOESM2_ESM.docx]

**Additional file 2**

Platform Ovid. Databases: MEDLINE(R), APA PsycInfo, Embase , HMIC Health Management Information Consortium, Social Policy and Practice

**Limits:** No filters, No date limits

| **Search terms** | |
| --- | --- |
| 1 | multimedia.ti,ab. |
| 2 | internet.ti,ab. |
| 3 | technolog*.ti,ab. |
| 4 | digital*.ti,ab. |
| 5 | computer*.ti,ab. |
| 6 | "ipad*".ti,ab. |
| 7 | "app".ti,ab. |
| 8 | "artificial intelligence".ti,ab. |
| 9 | robot*.ti,ab. |
| 10 | Whatsapp*.ti,ab. |
| 11 | facebook*.ti,ab. |
| 12 | twitter*.ti,ab. |
| 13 | skype*.ti,ab. |
| 14 | cellphone*.ti,ab. |
| 15 | smartphone*.ti,ab. |
| 16 | "virtual realit*".ti,ab. |
| 17 | software*.ti. |
| 18 | "PC".ti,ab. |
| 19 | "online*".ti,ab. |
| 20 | "console*".ti,ab. |
| 21 | "touchscreen".ti,ab. |
| 22 | "touch screen".ti,ab. |
| 23 | "tablet computer".ti,ab. |
| 24 | "tablet device".ti,ab. |
| 25 | "alexa".ti,ab. |
| 26 | "AI".ti,ab. |
| 27 | "smart phone*".ti,ab. |
| 28 | "mobile *phone*".ti,ab. |
| 29 | "virtual".ti. |
| 30 | Snoezelen*.ab,ti. |
| 31 | electronic*.ti,ab. |
| 32 | "social media".ti,ab. |
| 33 | "wireless".ab,ti. |
| 34 | Web-based.ab,ti. |
| 35 | Video.ti,ab. |
| 36 | "EMR".ab,ti. |
| 37 | "EHR".ab,ti. |
| 38 | "electronic medical record".ab,ti. |
| 39 | "electronic health record".ab,ti. |
| 40 | gerontechnology.ab,ti. |
| 41 | "information technolog*".ab,ti. |
| 42 | "information communication technolog*".ab,ti. |
| 43 | "ICT".ab,ti. |
| 44 | "leisure device*".ab,ti. |
| 45 | "social device*".ab,ti. |
| 46 | Playlist*.ab,ti. |
| 47 | information science/ or communication/ or social media/ or telecommunications/ or electronic mail/ or television/ or videoconferencing/ or wireless technology/ or computing methodologies/ or information technology/ |
| 48 | communication/ or "cell phone use"/ |
| 49 | Internet/ |
| 50 | computer.mp. or Computers/ |
| 51 | software.mp. or Software/ |
| 52 | Robotics/ or robot.mp. |
| 53 | "“care home*”".ab,ti. |
| 54 | "“nursing home*”".ab,ti. |
| 55 | "“residential home*”".ab,ti. |
| 56 | "dement* home*".ab,ti. |
| 57 | "assisted living facilit*".ab,ti. |
| 58 | "residential aged care".ab,ti. |
| 59 | "residential facili*".ab,ti. |
| 60 | ("residential care home*" or "dement*care home*").ab,ti. |
| 61 | "nursing facili*".ab,ti. |
| 62 | "long term care".ab,ti. |
| 63 | "long-term care".ab,ti. |
| 64 | "LTC".ab,ti. |
| 65 | "nursing cent*".ab,ti. |
| 66 | "old* people* home".ab,ti. |
| 67 | "home* for the aged".ab,ti. |
| 68 | "home* for the elderly".ab,ti. |
| 69 | "old* age home".ab,ti. |
| 70 | "old* age facil*".ab,ti. |
| 71 | "retirement home*".ab,ti. |
| 72 | exp assisted living facilities/ or exp homes for the aged/ |
| 73 | exp nursing homes/ or intermediate care facilities/ or skilled nursing facilities/ |
| 74 | exp Residential Facilities/ or residential care home.mp. or exp Homes for the Aged/ |
| 75 | dement*.ab,ti. |
| 76 | alzheimer*.ab,ti. |
| 77 | creutzfeldt-jakob*.ab,ti. |
| 78 | "frontotemporal lobar degeneration".ab,ti. |
| 79 | "pick* disease".ab,ti. |
| 80 | huntington.ab,ti. |
| 81 | "lewy body".ab,ti. |
| 82 | dementia.mp. or exp Dementia, Vascular/ or exp AIDS Dementia Complex/ or exp Dementia/ or exp Frontotemporal Dementia/ or exp Dementia, Multi-Infarct/ |
| 83 | "sociali*".ab,ti. |
| 84 | "social* connected*".ab,ti. |
| 85 | "social* interaction*".ab,ti. |
| 86 | "social* aliena*".ab,ti. |
| 87 | "feel* alone* ".ab,ti. |
| 88 | lonel*.ab,ti. |
| 89 | disconnect*.ab,ti. |
| 90 | "social* isolat*".ab,ti. |
| 91 | "social* engage*".ab,ti. |
| 92 | "social network".ab,ti. |
| 93 | "social* integrat*".ab,ti. |
| 94 | "social* partici*".ab,ti. |
| 95 | "social* activ*".ab,ti. |
| 96 | "community network*".ab,ti. |
| 97 | "social* contact".ab,ti. |
| 98 | "social* exclusion".ab,ti. |
| 99 | "social* compan*".ab,ti. |
| 100 | "social* communit*".ab,ti. |
| 101 | "social* group".ab,ti. |
| 102 | "social* environment".ab,ti. |
| 103 | activit*.ti,ab. |
| 104 | individuali*.ti,ab. |
| 105 | personali*.ti,ab. |
| 106 | tailor*.ti,ab. |
| 107 | "person cent*".ti,ab. |
| 108 | "person-cent*".ti,ab. |
| 109 | psychosocial.ti,ab. |
| 110 | non-pharmacological.ti,ab. |
| 111 | preference*.ab,ti. |
| 112 | meaningful.ab,ti. |
| 113 | recreation*.ab,ti. |
| 114 | occupation.ab,ti. |
| 115 | adaptation.ab,ti. |
| 116 | purposeful.ab,ti. |
| 117 | remin*.ab,ti. |
| 118 | lifestor*.ab,ti. |
| 119 | "life stor*".ab,ti. |
| 120 | music*.ab,ti. |
| 121 | leisure.ab,ti. |
| 122 | snoezelen*.ab,ti. |
| 123 | Namaste.ab,ti. |
| 124 | Patient-Centered Care/ or person centred.mp. |
| 125 | social isolation/ or social marginalization/ or socialization/ |
| 126 | Recreation Therapy/ |
| 127 | Loneliness/ or lonely.mp. |
| 128 | Leisure Activities/ |
| 129 | non-pharmacological.mp. |
| 130 | psychosocial.mp. |
| 131 | 1 or 2 or 3 or 4 or 5 or 6 or 7 or 8 or 9 or 10 or 11 or 12 or 13 or 14 or 15 or 16 or 17 or 18 or 19 or 20 or 21 or 22 or 23 or 24 or 25 or 26 or 27 or 28 or 29 or 30 or 31 or 32 or 33 or 34 or 35 or 36 or 37 or 38 or 39 or 40 or 41 or 42 or 43 or 44 or 45 or 46 or 47 or 48 or 49 or 50 or 51 or 52 |
| 132 | 53 or 54 or 55 or 56 or 57 or 58 or 59 or 60 or 61 or 62 or 63 or 64 or 65 or 66 or 67 or 68 or 69 or 70 or 71 or 72 or 73 or 74 |
| 133 | 75 or 76 or 77 or 78 or 79 or 80 or 81 or 82 |
| 134 | 83 or 84 or 85 or 86 or 87 or 88 or 89 or 90 or 91 or 92 or 93 or 94 or 95 or 96 or 97 or 98 or 99 or 100 or 101 or 102 or 103 or 104 or 105 or 106 or 107 or 108 or 109 or 110 or 111 or 112 or 113 or 114 or 115 or 116 or 117 or 118 or 119 or 120 or 121 or 122 or 123 or 124 or 125 or 126 or 127 or 128 or 129 or 130 |
| 135 | 131 and 132 and 133 and 134 |
| 136 | Deduplicate |
