# Supplementary material for: Digital technologies to support people living with dementia in the care home setting to engage in meaningful occupations: protocol for a scoping review
Source: Syst Rev. 2021 Jun 21;10:179. doi: 10.1186/s13643-021-01715-4 (PMC8214930; doi:10.1186/s13643-021-01715-4)
Supplement: Supplementary file 3 — Additional file 3. Charting form. [file 13643_2021_1715_MOESM3_ESM.docx]

**Additional file 3: Charting form**

| **Review summary** | | |
| --- | --- | --- |
| Scoping review title | Digital technologies to support people living with dementia in the care home setting to engage in meaningful occupations. | |
| Research questions | “What types of digital technologies have been used to support the provision of meaningful occupation for PLWD in the care home setting?”  “What are the reported mechanisms of action, methods of delivery, facilitators and barriers?” | |
| Objectives | 1. Systematically map the published research on digital technologies that have been used to support the provision of meaningful occupation. 2. Summarise the interventions; mechanism of action, components and delivery in the care home setting. 3. Identify reported facilitators and barriers to implementation. | |
| **Reference** | | |
| Authors, year of publication | |  |
| Title, Journal | |  |
| Country | |  |
| Year study conducted | |  |
| Contact with author(s) | |  |
| **Aim/research question/objectives** | |  |
| **Study design** | |  |
| Intervention | |  |
| Comparator | |  |
| Duration of intervention | |  |
| Duration of follow-up | |  |
| Measurement stages (eg. At baseline, 1 week) | |  |
| **Participants** | | |
| Sample size | |  |
| Age | |  |
| Gender | |  |
| Diagnosis of dementia | |  |
| Severity of cognitive impairment | |  |
| Co-morbidities (e.g. Frailty, health conditions) | |  |
| Ethnicity | |  |
| Socio-economic group | |  |
| Participant attrition | |  |
| Other characteristics | |  |
| **Setting** | | |
| Description of care homes (e.g. Nursing, residential) | |  |
| Number of care homes included | |  |
| Types and number of staff (e.g. Qualified nurses, care assistants) | |  |
| Other characteristics (eg. Country, rural/urban, specialist facility, state owned/private, independent/group, size by number of beds) | |  |
| **Digital technology** | |  |
| Description of the technology | |  |
| Generic or developed specifically | |  |
| Development stages of the technology (e.g. End user involvement, PPI) | |  |
| Components | |  |
| Intended user and whether a user centred design considered | |  |
| Characteristics of the interface | |  |
| How was it delivered (e.g. Care staff, residents, researcher) | |  |
| Training | |  |
| Technology support | |  |
| Requirements (e.g. Internet access, computers) | |  |
| Hypothesised mechanism of action | |  |
| **Meaningful occupation** | |  |
| How did the technology support meaningful occupation (e.g. Personalised, social engagement, supports care staff) | |  |
| Information utilised to ensure its meaningful (e.g. Life history, preferences, functional abilities) | |  |
| **Outcomes** | |  |
| Measures assessed | |  |
| Results | |  |
| Conclusions | |  |
| Unanticipated outcomes | |  |
| **Barriers** | |  |
| To implementation | |  |
| To using the technology (e.g. design, accessibility) | |  |
| Perceptions | |  |
| **Facilitators** | |  |
| To implementation | |  |
| To using the technology (e.g. design, accessibility) | |  |
| Perceptions | |  |
